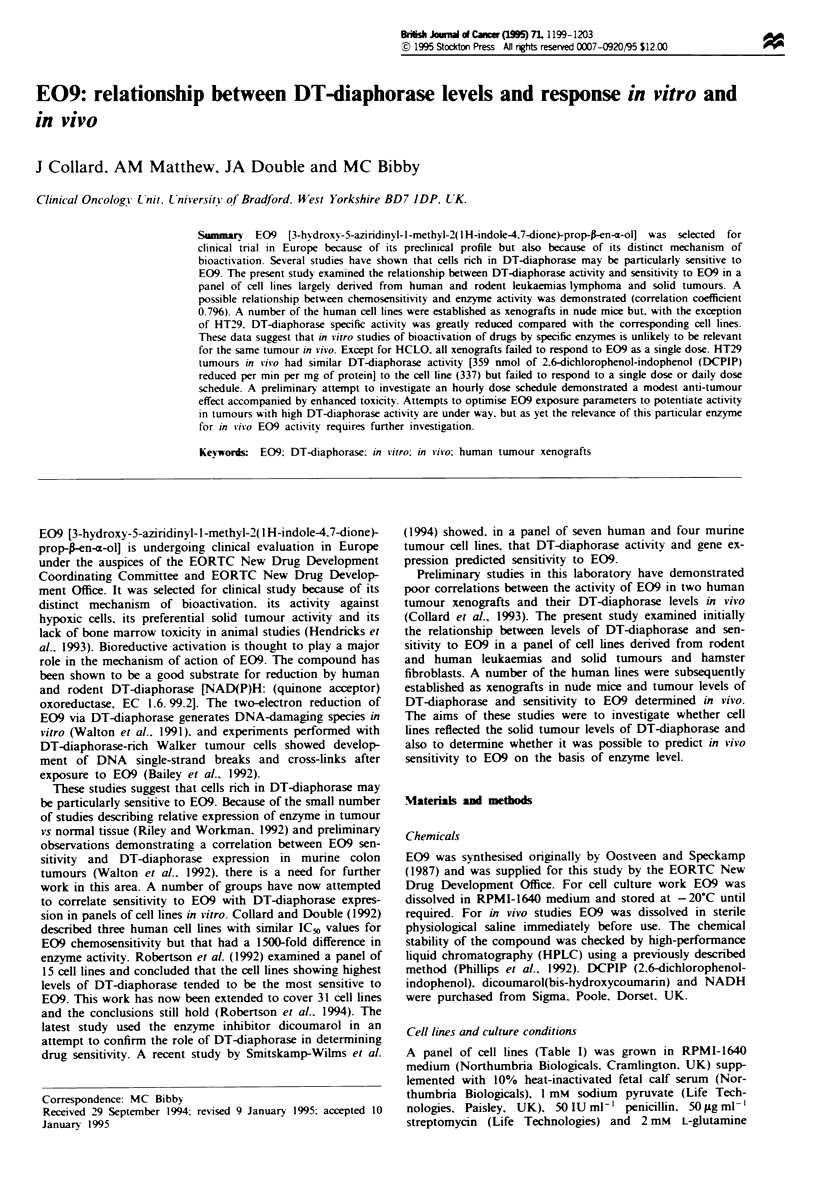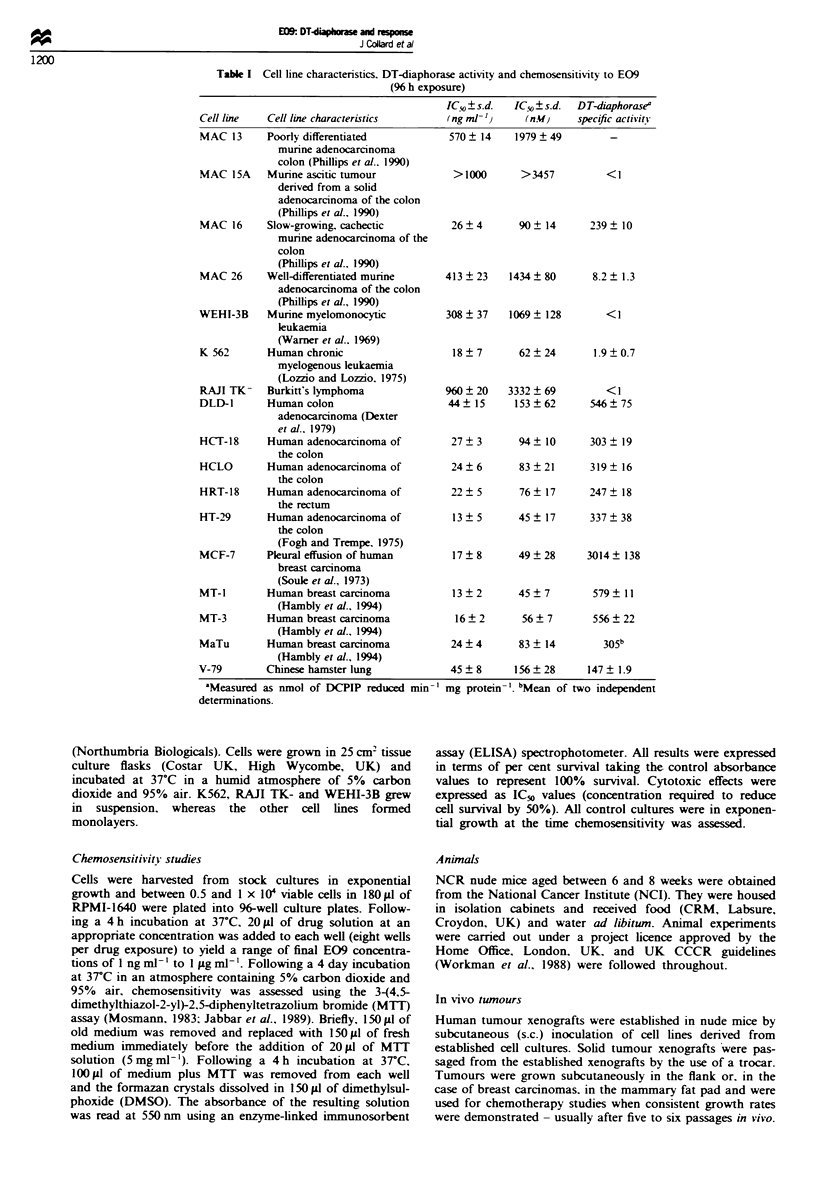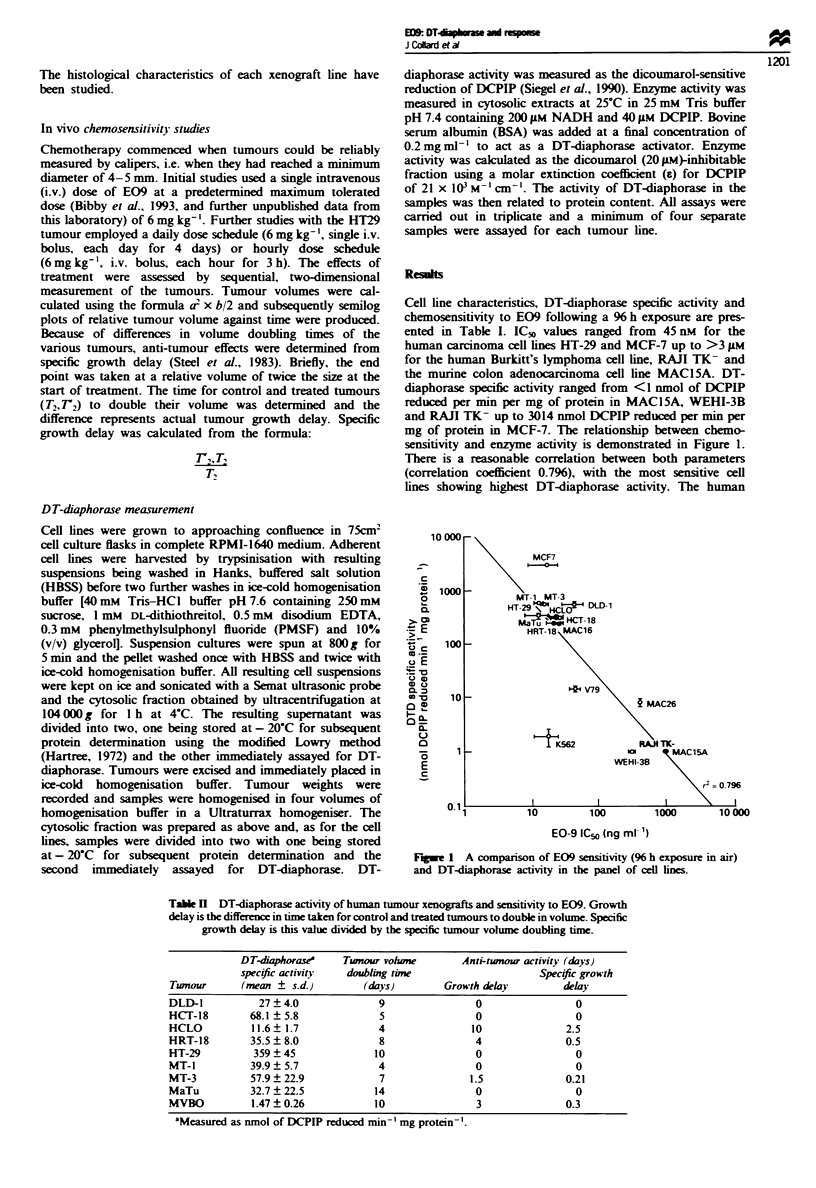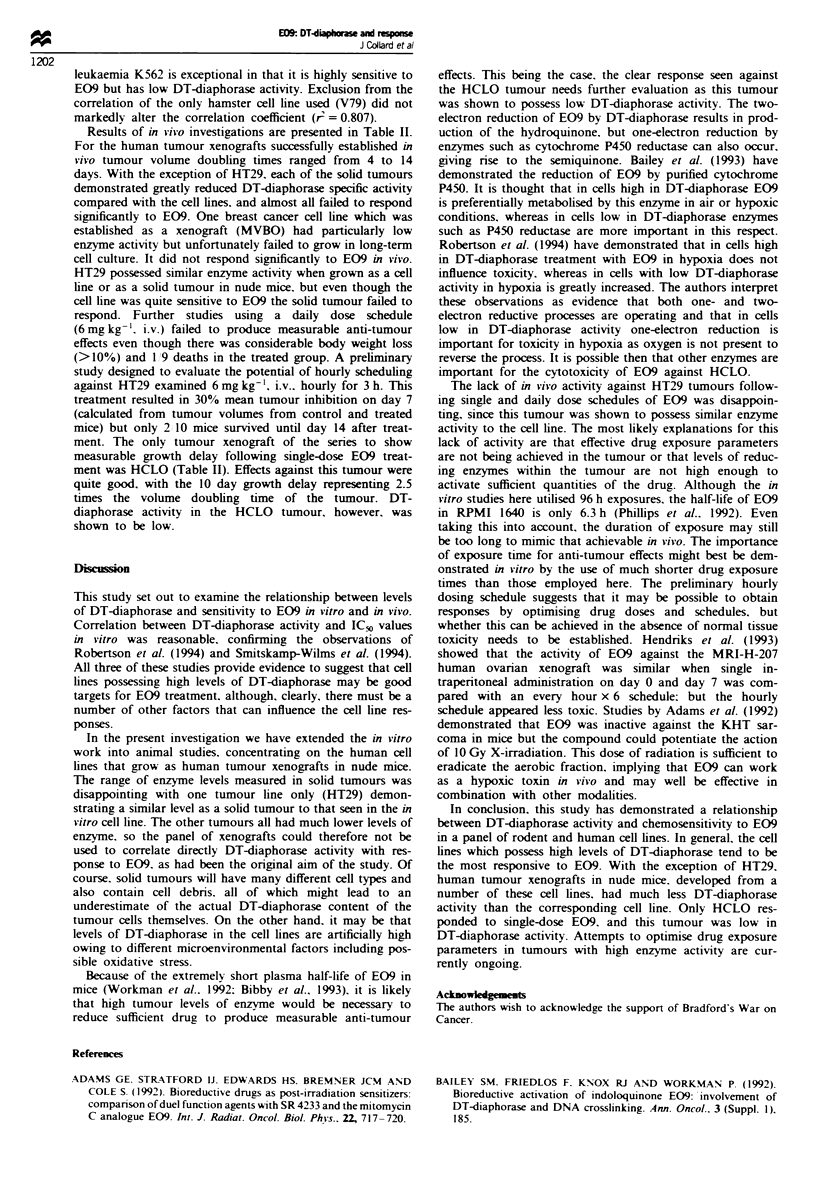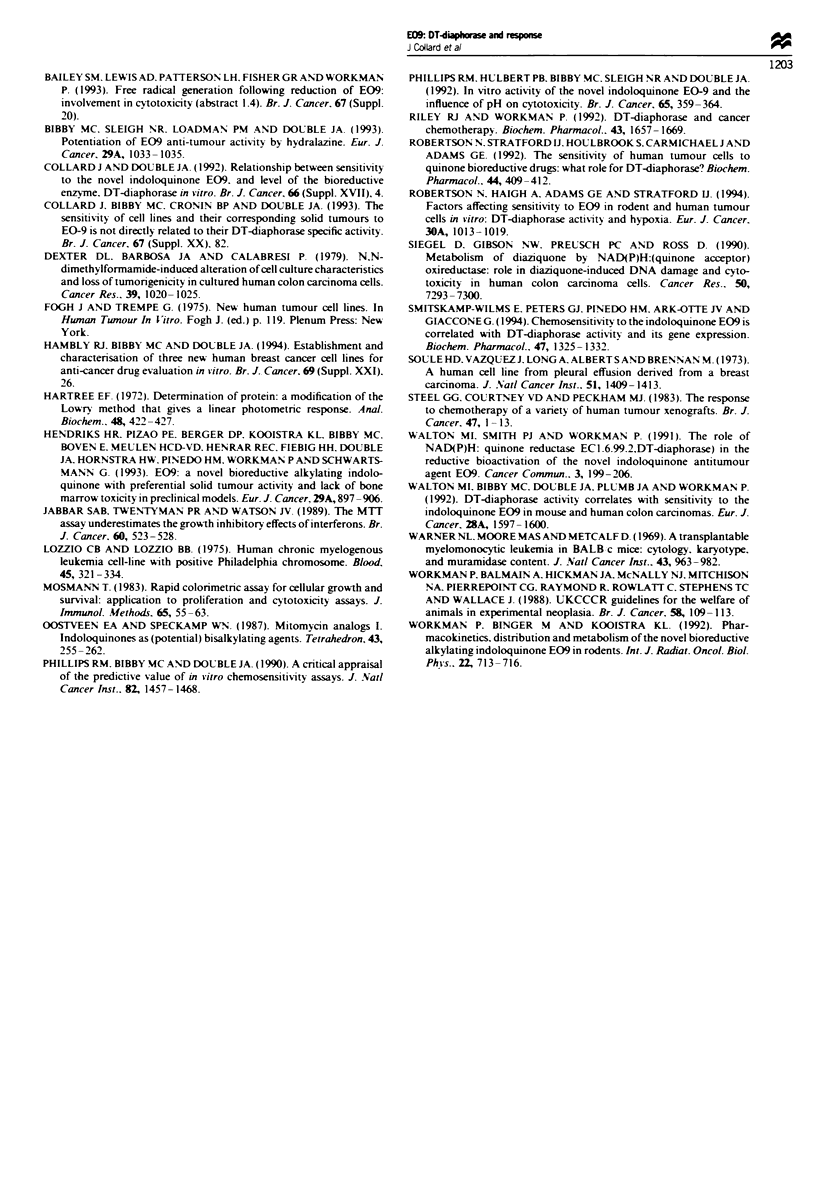# EO9: relationship between DT-diaphorase levels and response in vitro and in vivo.

**DOI:** 10.1038/bjc.1995.233

**Published:** 1995-06

**Authors:** J. Collard, A. M. Matthew, J. A. Double, M. C. Bibby

**Affiliations:** Clinical Oncology Unit, University of Bradford, West Yorkshire, UK.

## Abstract

EO9 [3-hydroxy-5-aziridinyl-1-methyl-2(1H-indole-4,7-dione)-prop-beta-en- alpha-ol] was selected for clinical trial in Europe because of its preclinical profile but also because of its distinct mechanism of bioactivation. Several studies have shown that cells rich in DT-diaphorase may be particularly sensitive to EO9. The present study examined the relationship between DT-diaphorase activity and sensitivity to EO9 in a panel of cell lines largely derived from human and rodent leukaemias/lymphoma and solid tumours. A possible relationship between chemosensitivity and enzyme activity was demonstrated (correlation coefficient 0.796). A number of the human cell lines were established as xenografts in nude mice but, with the exception of HT29, DT-diaphorase specific activity was greatly reduced compared with the corresponding cell lines. These data suggest that in vitro studies of bioactivation of drugs by specific enzymes is unlikely to be relevant for the same tumour in vivo. Except for HCLO, all xenografts failed to respond to EO9 as a single dose. HT29 tumours in vivo had similar DT-diaphorase activity [359 nmol of 2,6-dichlorophenol-indophenol (DCPIP) reduced per min per mg of protein] to the cell line (337) but failed to respond to a single dose or daily dose schedule. A preliminary attempt to investigate an hourly dose schedule demonstrated a modest anti-tumour effect accompanied by enhanced toxicity. Attempts to optimise EO9 exposure parameters to potentiate activity in tumours with high DT-diaphorase activity are under way, but as yet the relevance of this particular enzyme for in vivo EO9 activity requires further investigation.


British Journal of Cancer (1995) 71, 1199-1203

© 1995 Stockton Press All rights reserved 0007-0920/95 $12.00

### EO9: relationship between DT-diaphorase levels and response *in vitro* and *in vivo*

J Collard, AM Matthew, JA Double and MC Bibby

*Clinical Oncology Unit, University of Bradford, West Yorkshire BD7 1DP, UK.*

**Summary** EO9 [3-hydroxy-5-aziridinyl-1-methyl-2(1H-indole-4,7-dione)-prop-β-en-α-ol] was selected for clinical trial in Europe because of its preclinical profile but also because of its distinct mechanism of bioactivation. Several studies have shown that cells rich in DT-diaphorase may be particularly sensitive to EO9. The present study examined the relationship between DT-diaphorase activity and sensitivity to EO9 in a panel of cell lines largely derived from human and rodent leukaemias lymphoma and solid tumours. A possible relationship between chemosensitivity and enzyme activity was demonstrated (correlation coefficient 0.796). A number of the human cell lines were established as xenografts in nude mice but, with the exception of HT29, DT-diaphorase specific activity was greatly reduced compared with the corresponding cell lines. These data suggest that *in vitro* studies of bioactivation of drugs by specific enzymes is unlikely to be relevant for the same tumour *in vivo*. Except for HCLO, all xenografts failed to respond to EO9 as a single dose. HT29 tumours *in vivo* had similar DT-diaphorase activity [359 nmol of 2,6-dichlorophenol-indophenol (DCPIP) reduced per min per mg of protein] to the cell line (337) but failed to respond to a single dose or daily dose schedule. A preliminary attempt to investigate an hourly dose schedule demonstrated a modest anti-tumour effect accompanied by enhanced toxicity. Attempts to optimise EO9 exposure parameters to potentiate activity in tumours with high DT-diaphorase activity are under way, but as yet the relevance of this particular enzyme for *in vivo* EO9 activity requires further investigation.

**Keywords:** EO9; DT-diaphorase; *in vitro*; *in vivo*; human tumour xenografts

EO9 [3-hydroxy-5-aziridinyl-1-methyl-2(1H-indole-4,7-dione)-prop-β-en-α-ol] is undergoing clinical evaluation in Europe under the auspices of the EORTC New Drug Development Coordinating Committee and EORTC New Drug Development Office. It was selected for clinical study because of its distinct mechanism of bioactivation, its activity against hypoxic cells, its preferential solid tumour activity and its lack of bone marrow toxicity in animal studies (Hendricks *et al.*, 1993). Bioreductive activation is thought to play a major role in the mechanism of action of EO9. The compound has been shown to be a good substrate for reduction by human and rodent DT-diaphorase [NAD(P)H: (quinone acceptor) oxoreductase, EC 1.6. 99.2]. The two-electron reduction of EO9 via DT-diaphorase generates DNA-damaging species *in vitro* (Walton *et al.*, 1991), and experiments performed with DT-diaphorase-rich Walker tumour cells showed development of DNA single-strand breaks and cross-links after exposure to EO9 (Bailey *et al.*, 1992).

These studies suggest that cells rich in DT-diaphorase may be particularly sensitive to EO9. Because of the small number of studies describing relative expression of enzyme in tumour *vs* normal tissue (Riley and Workman, 1992) and preliminary observations demonstrating a correlation between EO9 sensitivity and DT-diaphorase expression in murine colon tumours (Walton *et al.*, 1992), there is a need for further work in this area. A number of groups have now attempted to correlate sensitivity to EO9 with DT-diaphorase expression in panels of cell lines *in vitro*. Collard and Double (1992) described three human cell lines with similar $IC_{50}$ values for EO9 chemosensitivity but that had a 1500-fold difference in enzyme activity. Robertson *et al.* (1992) examined a panel of 15 cell lines and concluded that the cell lines showing highest levels of DT-diaphorase tended to be the most sensitive to EO9. This work has now been extended to cover 31 cell lines and the conclusions still hold (Robertson *et al.*, 1994). The latest study used the enzyme inhibitor dicoumarol in an attempt to confirm the role of DT-diaphorase in determining drug sensitivity. A recent study by Smitskamp-Wilms *et al.* (1994) showed, in a panel of seven human and four murine tumour cell lines, that DT-diaphorase activity and gene expression predicted sensitivity to EO9.

Preliminary studies in this laboratory have demonstrated poor correlations between the activity of EO9 in two human tumour xenografts and their DT-diaphorase levels *in vivo* (Collard *et al.*, 1993). The present study examined initially the relationship between levels of DT-diaphorase and sensitivity to EO9 in a panel of cell lines derived from rodent and human leukaemias and solid tumours and hamster fibroblasts. A number of the human lines were subsequently established as xenografts in nude mice and tumour levels of DT-diaphorase and sensitivity to EO9 determined *in vivo*. The aims of these studies were to investigate whether cell lines reflected the solid tumour levels of DT-diaphorase and also to determine whether it was possible to predict *in vivo* sensitivity to EO9 on the basis of enzyme level.

### Materials and methods

### *Chemicals*

EO9 was synthesised originally by Oostveen and Speckamp (1987) and was supplied for this study by the EORTC New Drug Development Office. For cell culture work EO9 was dissolved in RPMI-1640 medium and stored at −20°C until required. For *in vivo* studies EO9 was dissolved in sterile physiological saline immediately before use. The chemical stability of the compound was checked by high-performance liquid chromatography (HPLC) using a previously described method (Phillips *et al.*, 1992). DCPIP (2,6-dichlorophenol-indophenol), dicoumarol(bis-hydroxycoumarin) and NADH were purchased from Sigma, Poole, Dorset, UK.

### *Cell lines and culture conditions*

A panel of cell lines (Table I) was grown in RPMI-1640 medium (Northumbria Biologicals, Cramlington, UK) supplemented with 10% heat-inactivated fetal calf serum (Northumbria Biologicals), 1 mM sodium pyruvate (Life Technologies, Paisley, UK), 50 IU $ml^{-1}$ penicillin, 50 $\mu g$ $ml^{-1}$ streptomycin (Life Technologies) and 2 mM L-glutamine

Correspondence: MC Bibby
Received 29 September 1994; revised 9 January 1995; accepted 10 January 1995

**Table I** Cell line characteristics, DT-diaphorase activity and chemosensitivity to EO9 (96 h exposure)

| *Cell line* | *Cell line characteristics* | *$IC_{50}$ ± s.d. (ng ml$^{-1}$)* | *$IC_{50}$ ± s.d. (nM)* | *DT-diaphorase[a] specific activity* |
|---|---|---|---|---|
| MAC 13 | Poorly differentiated murine adenocarcinoma colon (Phillips *et al.*, 1990) | 570 ± 14 | 1979 ± 49 | – |
| MAC 15A | Murine ascitic tumour derived from a solid adenocarcinoma of the colon (Phillips *et al.*, 1990) | >1000 | >3457 | <1 |
| MAC 16 | Slow-growing, cachectic murine adenocarcinoma of the colon (Phillips *et al.*, 1990) | 26 ± 4 | 90 ± 14 | 239 ± 10 |
| MAC 26 | Well-differentiated murine adenocarcinoma of the colon (Phillips *et al.*, 1990) | 413 ± 23 | 1434 ± 80 | 8.2 ± 1.3 |
| WEHI-3B | Murine myelomonocytic leukaemia (Warner *et al.*, 1969) | 308 ± 37 | 1069 ± 128 | <1 |
| K 562 | Human chronic myelogenous leukaemia (Lozzio and Lozzio, 1975) | 18 ± 7 | 62 ± 24 | 1.9 ± 0.7 |
| RAJI TK$^-$ | Burkitt's lymphoma | 960 ± 20 | 3332 ± 69 | <1 |
| DLD-1 | Human colon adenocarcinoma (Dexter *et al.*, 1979) | 44 ± 15 | 153 ± 62 | 546 ± 75 |
| HCT-18 | Human adenocarcinoma of the colon | 27 ± 3 | 94 ± 10 | 303 ± 19 |
| HCLO | Human adenocarcinoma of the colon | 24 ± 6 | 83 ± 21 | 319 ± 16 |
| HRT-18 | Human adenocarcinoma of the rectum | 22 ± 5 | 76 ± 17 | 247 ± 18 |
| HT-29 | Human adenocarcinoma of the colon (Fogh and Trempe, 1975) | 13 ± 5 | 45 ± 17 | 337 ± 38 |
| MCF-7 | Pleural effusion of human breast carcinoma (Soule *et al.*, 1973) | 17 ± 8 | 49 ± 28 | 3014 ± 138 |
| MT-1 | Human breast carcinoma (Hambly *et al.*, 1994) | 13 ± 2 | 45 ± 7 | 579 ± 11 |
| MT-3 | Human breast carcinoma (Hambly *et al.*, 1994) | 16 ± 2 | 56 ± 7 | 556 ± 22 |
| MaTu | Human breast carcinoma (Hambly *et al.*, 1994) | 24 ± 4 | 83 ± 14 | 305[b] |
| V-79 | Chinese hamster lung | 45 ± 8 | 156 ± 28 | 147 ± 1.9 |

[a]Measured as nmol of DCPIP reduced min$^{-1}$ mg protein$^{-1}$. [b]Mean of two independent determinations.

(Northumbria Biologicals). Cells were grown in 25 cm$^2$ tissue culture flasks (Costar UK, High Wycombe, UK) and incubated at 37°C in a humid atmosphere of 5% carbon dioxide and 95% air. K562, RAJI TK- and WEHI-3B grew in suspension, whereas the other cell lines formed monolayers.

*Chemosensitivity studies*

Cells were harvested from stock cultures in exponential growth and between 0.5 and 1 × $10^4$ viable cells in 180 μl of RPMI-1640 were plated into 96-well culture plates. Following a 4 h incubation at 37°C, 20 μl of drug solution at an appropriate concentration was added to each well (eight wells per drug exposure) to yield a range of final EO9 concentrations of 1 ng ml$^{-1}$ to 1 μg ml$^{-1}$. Following a 4 day incubation at 37°C in an atmosphere containing 5% carbon dioxide and 95% air, chemosensitivity was assessed using the 3-(4,5-dimethylthiazol-2-yl)-2,5-diphenyltetrazolium bromide (MTT) assay (Mosmann, 1983; Jabbar *et al.*, 1989). Briefly, 150 μl of old medium was removed and replaced with 150 μl of fresh medium immediately before the addition of 20 μl of MTT solution (5 mg ml$^{-1}$). Following a 4 h incubation at 37°C, 100 μl of medium plus MTT was removed from each well and the formazan crystals dissolved in 150 μl of dimethylsulphoxide (DMSO). The absorbance of the resulting solution was read at 550 nm using an enzyme-linked immunosorbent assay (ELISA) spectrophotometer. All results were expressed in terms of per cent survival taking the control absorbance values to represent 100% survival. Cytotoxic effects were expressed as $IC_{50}$ values (concentration required to reduce cell survival by 50%). All control cultures were in exponential growth at the time chemosensitivity was assessed.

*Animals*

NCR nude mice aged between 6 and 8 weeks were obtained from the National Cancer Institute (NCI). They were housed in isolation cabinets and received food (CRM, Labsure, Croydon, UK) and water *ad libitum*. Animal experiments were carried out under a project licence approved by the Home Office, London, UK, and UK CCCR guidelines (Workman *et al.*, 1988) were followed throughout.

In vivo *tumours*

Human tumour xenografts were established in nude mice by subcutaneous (s.c.) inoculation of cell lines derived from established cell cultures. Solid tumour xenografts were passaged from the established xenografts by the use of a trocar. Tumours were grown subcutaneously in the flank or, in the case of breast carcinomas, in the mammary fat pad and were used for chemotherapy studies when consistent growth rates were demonstrated – usually after five to six passages *in vivo*.

The histological characteristics of each xenograft line have been studied.

### In vivo *chemosensitivity studies*

Chemotherapy commenced when tumours could be reliably measured by calipers, i.e. when they had reached a minimum diameter of 4–5 mm. Initial studies used a single intravenous (i.v.) dose of EO9 at a predetermined maximum tolerated dose (Bibby *et al.*, 1993, and further unpublished data from this laboratory) of 6 mg $kg^{-1}$. Further studies with the HT29 tumour employed a daily dose schedule (6 mg $kg^{-1}$, single i.v. bolus, each day for 4 days) or hourly dose schedule (6 mg $kg^{-1}$, i.v. bolus, each hour for 3 h). The effects of treatment were assessed by sequential, two-dimensional measurement of the tumours. Tumour volumes were calculated using the formula $a^2 \times b/2$ and subsequently semilog plots of relative tumour volume against time were produced. Because of differences in volume doubling times of the various tumours, anti-tumour effects were determined from specific growth delay (Steel *et al.*, 1983). Briefly, the end point was taken at a relative volume of twice the size at the start of treatment. The time for control and treated tumours ($T_2$,$T'_2$) to double their volume was determined and the difference represents actual tumour growth delay. Specific growth delay was calculated from the formula:

$$\frac{T'_2,T_2}{T_2}$$

### *DT-diaphorase measurement*

Cell lines were grown to approaching confluence in 75cm² cell culture flasks in complete RPMI-1640 medium. Adherent cell lines were harvested by trypsinisation with resulting suspensions being washed in Hanks, buffered salt solution (HBSS) before two further washes in ice-cold homogenisation buffer [40 mM Tris–HCl buffer pH 7.6 containing 250 mM sucrose, 1 mM DL-dithiothreitol, 0.5 mM disodium EDTA, 0.3 mM phenylmethylsulphonyl fluoride (PMSF) and 10% (v/v) glycerol]. Suspension cultures were spun at 800 *g* for 5 min and the pellet washed once with HBSS and twice with ice-cold homogenisation buffer. All resulting cell suspensions were kept on ice and sonicated with a Semat ultrasonic probe and the cytosolic fraction obtained by ultracentrifugation at 104 000 *g* for 1 h at 4°C. The resulting supernatant was divided into two, one being stored at – 20°C for subsequent protein determination using the modified Lowry method (Hartree, 1972) and the other immediately assayed for DT-diaphorase. Tumours were excised and immediately placed in ice-cold homogenisation buffer. Tumour weights were recorded and samples were homogenised in four volumes of homogenisation buffer in a Ultraturrax homogeniser. The cytosolic fraction was prepared as above and, as for the cell lines, samples were divided into two with one being stored at – 20°C for subsequent protein determination and the second immediately assayed for DT-diaphorase. DT-diaphorase activity was measured as the dicoumarol-sensitive reduction of DCPIP (Siegel *et al.*, 1990). Enzyme activity was measured in cytosolic extracts at 25°C in 25 mM Tris buffer pH 7.4 containing 200 μM NADH and 40 μM DCPIP. Bovine serum albumin (BSA) was added at a final concentration of 0.2 mg $ml^{-1}$ to act as a DT-diaphorase activator. Enzyme activity was calculated as the dicoumarol (20 μM)-inhibitable fraction using a molar extinction coefficient (ε) for DCPIP of $21 \times 10^3\ M^{-1}\ cm^{-1}$. The activity of DT-diaphorase in the samples was then related to protein content. All assays were carried out in triplicate and a minimum of four separate samples were assayed for each tumour line.

### Results

Cell line characteristics, DT-diaphorase specific activity and chemosensitivity to EO9 following a 96 h exposure are presented in Table I. $IC_{50}$ values ranged from 45 nM for the human carcinoma cell lines HT-29 and MCF-7 up to >3 μM for the human Burkitt's lymphoma cell line, RAJI $TK^-$ and the murine colon adenocarcinoma cell line MAC15A. DT-diaphorase specific activity ranged from <1 nmol of DCPIP reduced per min per mg of protein in MAC15A, WEHI-3B and RAJI $TK^-$ up to 3014 nmol DCPIP reduced per min per mg of protein in MCF-7. The relationship between chemosensitivity and enzyme activity is demonstrated in Figure 1. There is a reasonable correlation between both parameters (correlation coefficient 0.796), with the most sensitive cell lines showing highest DT-diaphorase activity. The human

**Figure 1** A comparison of EO9 sensitivity (96 h exposure in air) and DT-diaphorase activity in the panel of cell lines.

**Table II** DT-diaphorase activity of human tumour xenografts and sensitivity to EO9. Growth delay is the difference in time taken for control and treated tumours to double in volume. Specific growth delay is this value divided by the specific tumour volume doubling time.

| *Tumour* | *DT-diaphorase[a] specific activity (mean ± s.d.)* | *Tumour volume doubling time (days)* | *Anti-tumour activity (days)* Growth delay | *Specific growth delay* |
|---|---|---|---|---|
| DLD-1 | 27 ± 4.0 | 9 | 0 | 0 |
| HCT-18 | 68.1 ± 5.8 | 5 | 0 | 0 |
| HCLO | 11.6 ± 1.7 | 4 | 10 | 2.5 |
| HRT-18 | 35.5 ± 8.0 | 8 | 4 | 0.5 |
| HT-29 | 359 ± 45 | 10 | 0 | 0 |
| MT-1 | 39.9 ± 5.7 | 4 | 0 | 0 |
| MT-3 | 57.9 ± 22.9 | 7 | 1.5 | 0.21 |
| MaTu | 32.7 ± 22.5 | 14 | 0 | 0 |
| MVBO | 1.47 ± 0.26 | 10 | 3 | 0.3 |

[a]Measured as nmol of DCPIP reduced $min^{-1}$ mg $protein^{-1}$.

leukaemia K562 is exceptional in that it is highly sensitive to EO9 but has low DT-diaphorase activity. Exclusion from the correlation of the only hamster cell line used (V79) did not markedly alter the correlation coefficient ($r^2 = 0.807$).

Results of *in vivo* investigations are presented in Table II. For the human tumour xenografts successfully established *in vivo* tumour volume doubling times ranged from 4 to 14 days. With the exception of HT29, each of the solid tumours demonstrated greatly reduced DT-diaphorase specific activity compared with the cell lines, and almost all failed to respond significantly to EO9. One breast cancer cell line which was established as a xenograft (MVBO) had particularly low enzyme activity but unfortunately failed to grow in long-term cell culture. It did not respond significantly to EO9 *in vivo*. HT29 possessed similar enzyme activity when grown as a cell line or as a solid tumour in nude mice, but even though the cell line was quite sensitive to EO9 the solid tumour failed to respond. Further studies using a daily dose schedule (6 mg $kg^{-1}$, i.v.) failed to produce measurable anti-tumour effects even though there was considerable body weight loss (>10%) and 1/9 deaths in the treated group. A preliminary study designed to evaluate the potential of hourly scheduling against HT29 examined 6 mg $kg^{-1}$, i.v., hourly for 3 h. This treatment resulted in 30% mean tumour inhibition on day 7 (calculated from tumour volumes from control and treated mice) but only 2/10 mice survived until day 14 after treatment. The only tumour xenograft of the series to show measurable growth delay following single-dose EO9 treatment was HCLO (Table II). Effects against this tumour were quite good, with the 10 day growth delay representing 2.5 times the volume doubling time of the tumour. DT-diaphorase activity in the HCLO tumour, however, was shown to be low.

### Discussion

This study set out to examine the relationship between levels of DT-diaphorase and sensitivity to EO9 *in vitro* and *in vivo*. Correlation between DT-diaphorase activity and $IC_{50}$ values *in vitro* was reasonable, confirming the observations of Robertson *et al.* (1994) and Smitskamp-Wilms *et al.* (1994). All three of these studies provide evidence to suggest that cell lines possessing high levels of DT-diaphorase may be good targets for EO9 treatment, although, clearly, there must be a number of other factors that can influence the cell line responses.

In the present investigation we have extended the *in vitro* work into animal studies, concentrating on the human cell lines that grow as human tumour xenografts in nude mice. The range of enzyme levels measured in solid tumours was disappointing with one tumour line only (HT29) demonstrating a similar level as a solid tumour to that seen in the *in vitro* cell line. The other tumours all had much lower levels of enzyme, so the panel of xenografts could therefore not be used to correlate directly DT-diaphorase activity with response to EO9, as had been the original aim of the study. Of course, solid tumours will have many different cell types and also contain cell debris, all of which might lead to an underestimate of the actual DT-diaphorase content of the tumour cells themselves. On the other hand, it may be that levels of DT-diaphorase in the cell lines are artificially high owing to different microenvironmental factors including possible oxidative stress.

Because of the extremely short plasma half-life of EO9 in mice (Workman *et al.*, 1992; Bibby *et al.*, 1993), it is likely that high tumour levels of enzyme would be necessary to reduce sufficient drug to produce measurable anti-tumour effects. This being the case, the clear response seen against the HCLO tumour needs further evaluation as this tumour was shown to possess low DT-diaphorase activity. The two-electron reduction of EO9 by DT-diaphorase results in production of the hydroquinone, but one-electron reduction by enzymes such as cytochrome P450 reductase can also occur, giving rise to the semiquinone. Bailey *et al.* (1993) have demonstrated the reduction of EO9 by purified cytochrome P450. It is thought that in cells high in DT-diaphorase EO9 is preferentially metabolised by this enzyme in air or hypoxic conditions, whereas in cells low in DT-diaphorase enzymes such as P450 reductase are more important in this respect. Robertson *et al.* (1994) have demonstrated that in cells high in DT-diaphorase treatment with EO9 in hypoxia does not influence toxicity, whereas in cells with low DT-diaphorase activity in hypoxia is greatly increased. The authors interpret these observations as evidence that both one- and two-electron reductive processes are operating and that in cells low in DT-diaphorase activity one-electron reduction is important for toxicity in hypoxia as oxygen is not present to reverse the process. It is possible then that other enzymes are important for the cytotoxicity of EO9 against HCLO.

The lack of *in vivo* activity against HT29 tumours following single and daily dose schedules of EO9 was disappointing, since this tumour was shown to possess similar enzyme activity to the cell line. The most likely explanations for this lack of activity are that effective drug exposure parameters are not being achieved in the tumour or that levels of reducing enzymes within the tumour are not high enough to activate sufficient quantities of the drug. Although the *in vitro* studies here utilised 96 h exposures, the half-life of EO9 in RPMI 1640 is only 6.3 h (Phillips *et al.*, 1992). Even taking this into account, the duration of exposure may still be too long to mimic that achievable *in vivo*. The importance of exposure time for anti-tumour effects might best be demonstrated *in vitro* by the use of much shorter drug exposure times than those employed here. The preliminary hourly dosing schedule suggests that it may be possible to obtain responses by optimising drug doses and schedules, but whether this can be achieved in the absence of normal tissue toxicity needs to be established. Hendriks *et al.* (1993) showed that the activity of EO9 against the MRI-H-207 human ovarian xenograft was similar when single intraperitoneal administration on day 0 and day 7 was compared with an every hour × 6 schedule; but the hourly schedule appeared less toxic. Studies by Adams *et al.* (1992) demonstrated that EO9 was inactive against the KHT sarcoma in mice but the compound could potentiate the action of 10 Gy X-irradiation. This dose of radiation is sufficient to eradicate the aerobic fraction, implying that EO9 can work as a hypoxic toxin *in vivo* and may well be effective in combination with other modalities.

In conclusion, this study has demonstrated a relationship between DT-diaphorase activity and chemosensitivity to EO9 in a panel of rodent and human cell lines. In general, the cell lines which possess high levels of DT-diaphorase tend to be the most responsive to EO9. With the exception of HT29, human tumour xenografts in nude mice, developed from a number of these cell lines, had much less DT-diaphorase activity than the corresponding cell line. Only HCLO responded to single-dose EO9, and this tumour was low in DT-diaphorase activity. Attempts to optimise drug exposure parameters in tumours with high enzyme activity are currently ongoing.

**Acknowledgements**

The authors wish to acknowledge the support of Bradford's War on Cancer.

### References

ADAMS GE, STRATFORD IJ, EDWARDS HS, BREMNER JCM AND COLE S. (1992). Bioreductive drugs as post-irradiation sensitizers: comparison of duel function agents with SR 4233 and the mitomycin C analogue EO9. *Int. J. Radiat. Oncol. Biol. Phys.*, **22**, 717–720.

BAILEY SM, FRIEDLOS F, KNOX RJ AND WORKMAN P. (1992). Bioreductive activation of indoloquinone EO9: involvement of DT-diaphorase and DNA crosslinking. *Ann. Oncol.*, 3 (Suppl. 1), 185.

BAILEY SM. LEWIS AD. PATTERSON LH. FISHER GR AND WORKMAN P. (1993). Free radical generation following reduction of EO9: involvement in cytotoxicity (abstract 1.4). *Br. J. Cancer*, **67** (Suppl. 20).

BIBBY MC. SLEIGH NR. LOADMAN PM AND DOUBLE JA. (1993). Potentiation of EO9 anti-tumour activity by hydralazine. *Eur. J. Cancer*, **29A**, 1033-1035.

COLLARD J AND DOUBLE JA. (1992). Relationship between sensitivity to the novel indoloquinone EO9, and level of the bioreductive enzyme, DT-diaphorase *in vitro*. *Br. J. Cancer*, **66** (Suppl. XVII), 4.

COLLARD J. BIBBY MC. CRONIN BP AND DOUBLE JA. (1993). The sensitivity of cell lines and their corresponding solid tumours to EO-9 is not directly related to their DT-diaphorase specific activity. *Br. J. Cancer*, **67** (Suppl. XX), 82.

DEXTER DL. BARBOSA JA AND CALABRESI P. (1979). N,N-dimethylformamide-induced alteration of cell culture characteristics and loss of tumorigenicity in cultured human colon carcinoma cells. *Cancer Res.*, **39**, 1020-1025.

FOGH J AND TREMPE G. (1975). New human tumour cell lines. In *Human Tumour In Vitro*. Fogh J. (ed.) p. 119. Plenum Press: New York.

HAMBLY RJ. BIBBY MC AND DOUBLE JA. (1994). Establishment and characterisation of three new human breast cancer cell lines for anti-cancer drug evaluation *in vitro*. *Br. J. Cancer*, **69** (Suppl. XXI), 26.

HARTREE EF. (1972). Determination of protein: a modification of the Lowry method that gives a linear photometric response. *Anal. Biochem.*, **48**, 422-427.

HENDRIKS HR. PIZAO PE. BERGER DP. KOOISTRA KL. BIBBY MC. BOVEN E. MEULEN HCD-VD. HENRAR REC. FIEBIG HH. DOUBLE JA. HORNSTRA HW. PINEDO HM. WORKMAN P AND SCHWARTSMANN G. (1993). EO9: a novel bioreductive alkylating indoloquinone with preferential solid tumour activity and lack of bone marrow toxicity in preclinical models. *Eur. J. Cancer*, **29A**, 897-906.

JABBAR SAB. TWENTYMAN PR AND WATSON JV. (1989). The MTT assay underestimates the growth inhibitory effects of interferons. *Br. J. Cancer*, **60**, 523-528.

LOZZIO CB AND LOZZIO BB. (1975). Human chronic myelogenous leukemia cell-line with positive Philadelphia chromosome. *Blood*, **45**, 321-334.

MOSMANN T. (1983). Rapid colorimetric assay for cellular growth and survival: application to proliferation and cytotoxicity assays. *J. Immunol. Methods*, **65**, 55-63.

OOSTVEEN EA AND SPECKAMP WN. (1987). Mitomycin analogs I. Indoloquinones as (potential) bisalkylating agents. *Tetrahedron*, **43**, 255-262.

PHILLIPS RM. BIBBY MC AND DOUBLE JA. (1990). A critical appraisal of the predictive value of *in vitro* chemosensitivity assays. *J. Natl Cancer Inst.*, **82**, 1457-1468.

PHILLIPS RM. HULBERT PB. BIBBY MC. SLEIGH NR AND DOUBLE JA. (1992). In vitro activity of the novel indoloquinone EO-9 and the influence of pH on cytotoxicity. *Br. J. Cancer*, **65**, 359-364.

RILEY RJ AND WORKMAN P. (1992). DT-diaphorase and cancer chemotherapy. *Biochem. Pharmacol.*, **43**, 1657-1669.

ROBERTSON N. STRATFORD IJ. HOULBROOK S. CARMICHAEL J AND ADAMS GE. (1992). The sensitivity of human tumour cells to quinone bioreductive drugs: what role for DT-diaphorase? *Biochem. Pharmacol.*, **44**, 409-412.

ROBERTSON N. HAIGH A. ADAMS GE AND STRATFORD IJ. (1994). Factors affecting sensitivity to EO9 in rodent and human tumour cells *in vitro*: DT-diaphorase activity and hypoxia. *Eur. J. Cancer*, **30A**, 1013-1019.

SIEGEL D. GIBSON NW. PREUSCH PC AND ROSS D. (1990). Metabolism of diaziquone by NAD(P)H:(quinone acceptor) oxireductase: role in diaziquone-induced DNA damage and cytotoxicity in human colon carcinoma cells. *Cancer Res.*, **50**, 7293-7300.

SMITSKAMP-WILMS E. PETERS GJ. PINEDO HM. ARK-OTTE JV AND GIACCONE G. (1994). Chemosensitivity to the indoloquinone EO9 is correlated with DT-diaphorase activity and its gene expression. *Biochem. Pharmacol.*, **47**, 1325-1332.

SOULE HD. VAZQUEZ J. LONG A. ALBERT S AND BRENNAN M. (1973). A human cell line from pleural effusion derived from a breast carcinoma. *J. Natl Cancer Inst.*, **51**, 1409-1413.

STEEL GG. COURTNEY VD AND PECKHAM MJ. (1983). The response to chemotherapy of a variety of human tumour xenografts. *Br. J. Cancer*, **47**, 1-13.

WALTON MI. SMITH PJ AND WORKMAN P. (1991). The role of NAD(P)H: quinone reductase EC1.6.99.2,DT-diaphorase) in the reductive bioactivation of the novel indoloquinone antitumour agent EO9. *Cancer Commun.*, **3**, 199-206.

WALTON MI. BIBBY MC. DOUBLE JA. PLUMB JA AND WORKMAN P. (1992). DT-diaphorase activity correlates with sensitivity to the indoloquinone EO9 in mouse and human colon carcinomas. *Eur. J. Cancer*, **28A**, 1597-1600.

WARNER NL. MOORE MAS AND METCALF D. (1969). A transplantable myelomonocytic leukemia in BALB c mice: cytology, karyotype, and muramidase content. *J. Natl Cancer Inst.*, **43**, 963-982.

WORKMAN P. BALMAIN A. HICKMAN JA. McNALLY NJ. MITCHISON NA. PIERREPOINT CG. RAYMOND R. ROWLATT C. STEPHENS TC AND WALLACE J. (1988). UKCCCR guidelines for the welfare of animals in experimental neoplasia. *Br. J. Cancer*, **58**, 109-113.

WORKMAN P. BINGER M AND KOOISTRA KL. (1992). Pharmacokinetics, distribution and metabolism of the novel bioreductive alkylating indoloquinone EO9 in rodents. *Int. J. Radiat. Oncol. Biol. Phys.*, **22**, 713-716.